# The shift of soil microbial community induced by cropping sequence affect soil properties and crop yield

**DOI:** 10.3389/fmicb.2023.1095688

**Published:** 2023-02-16

**Authors:** Lei Sun, Shuang Wang, Manik Prabhu Narsing Rao, Yu Shi, Zheng-Han Lian, Pin-Jiao Jin, Wei Wang, Yu-Mei Li, Kang-Kang Wang, Aparna Banerjee, Xiao-Yang Cui, Dan Wei

**Affiliations:** ^1^Key Laboratory of Sustainable Forest Ecosystem Management-Ministry of Education, School of Forestry, Northeast Forestry University, Harbin, China; ^2^Heilongjiang Academy of Black Soil Conservation and Utilization, Harbin, China; ^3^Programa de Doctorado en Ciencias Aplicadas, Universidad Autónoma de Chile, Talca, Chile; ^4^State Key Laboratory of Crop Stress Adaptation and Improvement, School of Life Sciences, Henan University, Kaifeng, China; ^5^State Key Laboratory of Biocontrol, Guangdong Provincial Key Laboratory of Plant Resources and Southern Marine Science and Engineering Guangdong Laboratory (Zhuhai), School of Life Sciences, Sun Yat-sen University, Guangzhou, China; ^6^Centro de Investigación de Estudios Avanzados del Maule (CIEAM),Vicerrectoría de Investigación y Postgrado, Universidad Católica del Maule, Talca, Chile; ^7^Institute of Plant Nutrition and Resources, Beijing Academy of Agriculture and Forestry Sciences, Beijing, China

**Keywords:** crop rotation, soybean, wheat, maize, microbial community structure

## Abstract

Rational cropping maintains high soil fertility and a healthy ecosystem. Soil microorganism is the controller of soil fertility. Meanwhile, soil microbial communities also respond to different cropping patterns. The mechanisms by which biotic and abiotic factors were affected by different cropping sequences remain unclear in the major grain-producing regions of northeastern China. To evaluate the effects of different cropping sequences under conventional fertilization practices on soil properties, microbial communities, and crop yield, six types of plant cropping systems were performed, including soybean monoculture, wheat-soybean rotation, wheat-maize-soybean rotation, soybean-maize-maize rotation, maize-soybean-soybean rotation and maize monoculture. Our results showed that compared with the single cropping system, soybean and maize crop rotation in different combinations or sequences can increase soil total organic carbon and nutrients, and promote soybean and maize yield, especially using soybean-maize-maize and maize-soybean-soybean planting system. The 16S rRNA and internal transcribed spacer (ITS) amplicon sequencing showed that different cropping systems had different effects on bacterial and fungal communities. The bacterial and fungal communities of soybean monoculture were less diverse when compared to the other crop rotation planting system. Among the different cropping sequences, the number of observed bacterial species was greater in soybean-maize-maize planting setup and fungal species in maize-soybean-soybean planting setup. Some dominant and functional bacterial and fungal taxa in the rotation soils were observed. Network-based analysis suggests that bacterial phyla *Acidobacteria* and *Actinobacteria* while fungal phylum *Ascomycota* showed a positive correlation with other microbial communities. The phylogenetic investigation of communities by reconstruction of unobserved states (PICRUSt) result showed the presence of various metabolic pathways. Besides, the soybean-maize-maize significantly increased the proportion of some beneficial microorganisms in the soil and reduced the soil-borne animal and plant pathogens. These results warrant further investigation into the mechanisms driving responses of beneficial microbial communities and their capacity on improving soil fertility during legume cropping. The present study extends our understanding of how different crop rotations effect soil parameters, microbial diversity, and metabolic functions, and reveals the importance of crop rotation sequences. These findings could be used to guide decision-making from the microbial perspective for annual crop planting and soil management approaches.

## Introduction

China is the world’s largest soybean consumer and importer and is regarded as an important food crop, oil crop, and feed source (Yao et al., 2020). To attain overall soybean self-sufficiency in China, the government encourages farmers to cultivate more soybean crops (Tan et al., 2021). Statistical data showed that the world’s soybean production increased approximately 13-fold from 1961 to 2017 (Liu S. et al., 2020) Heihe city, in the Northeast of the country, has the largest area under soybean cultivation and the highest total yield in the country, the unique geographical and climatic location, soil composition, water, and air quality are all conducive to the production of green edible soybeans. However, continuous soybean planting caused soil acidification, aggravation of soil-borne diseases, a decrease in soil enzyme activity, and the accumulation of toxins in the soil (Yuan et al., 2021). To overcome this issue many plant cultivation models have been developed and among them crop rotation is one of the essential management approaches used by farmers (Neupane et al., 2021).

Crop rotation is the practice of planting different crops on the same land during successive growth/seeding cycles (Bullock, 1992; Yu et al., 2022). Since ancient times, crop rotation is a common management method used to improve soil nutrient and water availability, reduce weeds and pests, and improve the ecological and economic sustainability of cropping systems (Yang et al., 2021). Diversified crop rotation can even reduce the consequences of increased drought intensity and heatwaves even in drought circumstances (Bowles et al., 2020). Studies have shown that crop rotation enhances soybean yields. Using corn and winter wheat (with or without red clover) plants, Agomoh et al. (2021) investigated the effects of crop rotation on soybean production and discovered that soybeans grown in 3 years of rotations with corn and winter wheat provided the highest yields. Growing soybeans 1 year out of three in three-year cycles, such as corn–soybean–wheat, increased the yield of soybean (Lund et al., 1993). Studies also suggest that the practice of crop rotation helps to control the soybean cyst nematode (Sasser and Uzzell, 1991). In recent years, it has also been found that crop rotation effect subsurface microbial communities (Paungfoo-Lonhienne et al., 2017; Xie et al., 2020; Yu et al., 2021; Wang et al., 2022).

Microorganisms are an integral part of almost all soil, and some agronomic practices, such as fertilization and crop rotation, affect soil microbial communities and functions. Xie et al. (2022) found that the crop rotation stage strongly affected the soil microbial community structure and assembly compared to that of the fertilization regime. The cropping sequences within the rotation can change soil microbial communities and give favorable impacts for increased agronomic performance (Bolaji et al., 2021). Microorganisms have been shown to improve plant development by secreting metabolites, mobilizing nutrients, and alleviating biotic and abiotic stresses (Tahir et al., 2017; Bolaji et al., 2021). Crop rotation sequences appear to have a direct impact on the structure of microbial communities associated with soil and plants (Zhang et al., 2019; Neupane et al., 2021). The most diverse crop rotation showed the most diverse and active soil microbial biota (D’Acunto et al., 2018). Crop rotations can improve disease suppression capacity by influencing soil bacterial composition or increasing the quantity of disease-suppressive microorganisms (Peralta et al., 2018). Previous studies have shown that crop rotation strategies changed microbial communities over time due to cropping sequence practices (Bolaji et al., 2021; Meier et al., 2021). In wheat-soybean rotation, soybean planting increased the relative abundance of *Firmicutes* and *Glomeromycota* (Guo et al., 2020). Although there have been numerous studies on soybean crop rotation management, the effects of crop rotation order on soil microbial communities under conventional fertilization methods for different food crops, including soybean, are not well understood.

The choice of crop planting sequence is often related to the farmer’s goal looking to achieve with the rotation, which could be weed management, pest and disease control, increasing available nitrogen in the soil, controlling soil erosion, improving soil structure, and increasing economic benefits (Boincean and Dent, 2019). The local department of agriculture of Heilongjiang has also introduced a pilot scheme for crop rotation and fallow, including the “three-three system” of crop rotation (corn-soybean-wheat, corn-soybean-potato, corn-soybean-grain, etc.), and the “two-two system” of crop rotation (corn-soybean, corn-wheat, corn-potato, and so on) allowed.^1^

In the present study, six planting sequence setups were tested over five years in northeastern China. Planting 1 setup includes only soybean, planting 2 setup includes wheat and soybean, planting 3 set up includes wheat, maize, and soybean, planting 4 setup includes soybean and maize, planting 5 setup includes maize and soybean, and planting 6 setup includes only maize. Marker-based Illumina sequencing and bioinformatics analyses were carried out to understand cropping sequence and its effect on soil properties and crop yields-associated microbial communities. Our objectives were to explore some scientific questions: (i) does the planting sequence effect soil microorganisms? (ii) If so, are there core beneficial microorganisms that could increase crop yields? The answer to these questions can provide recommendations for reasonable crop rotation for local farmers and agricultural managers.

## Materials and methods

### Experimental setup and fertilization management

In the spring of 2012, the field positioning experiment of the new rotation system, wheat, maize and soybean was established in Sinograin’s agriculture demonstration zone (E 49°33′35″, N 125°27′5″, 225 m a.s.l.; soil type: dark brown soil) of Heihe city, China. The site lies within mid temperate semi-humid continental monsoon climate, characterized by low annual average temperature, and a short frost-free period that follows dry, long, and cold winters. In the last four decades, the annual precipitation average was 481 mm, while the mean annual temperature was 0.5°C, with a minimum and maximum monthly average of −29.2°C and 26.6°C, respectively. In this climate, each grain crop can only be planted one season a year.

**Figure 1:**
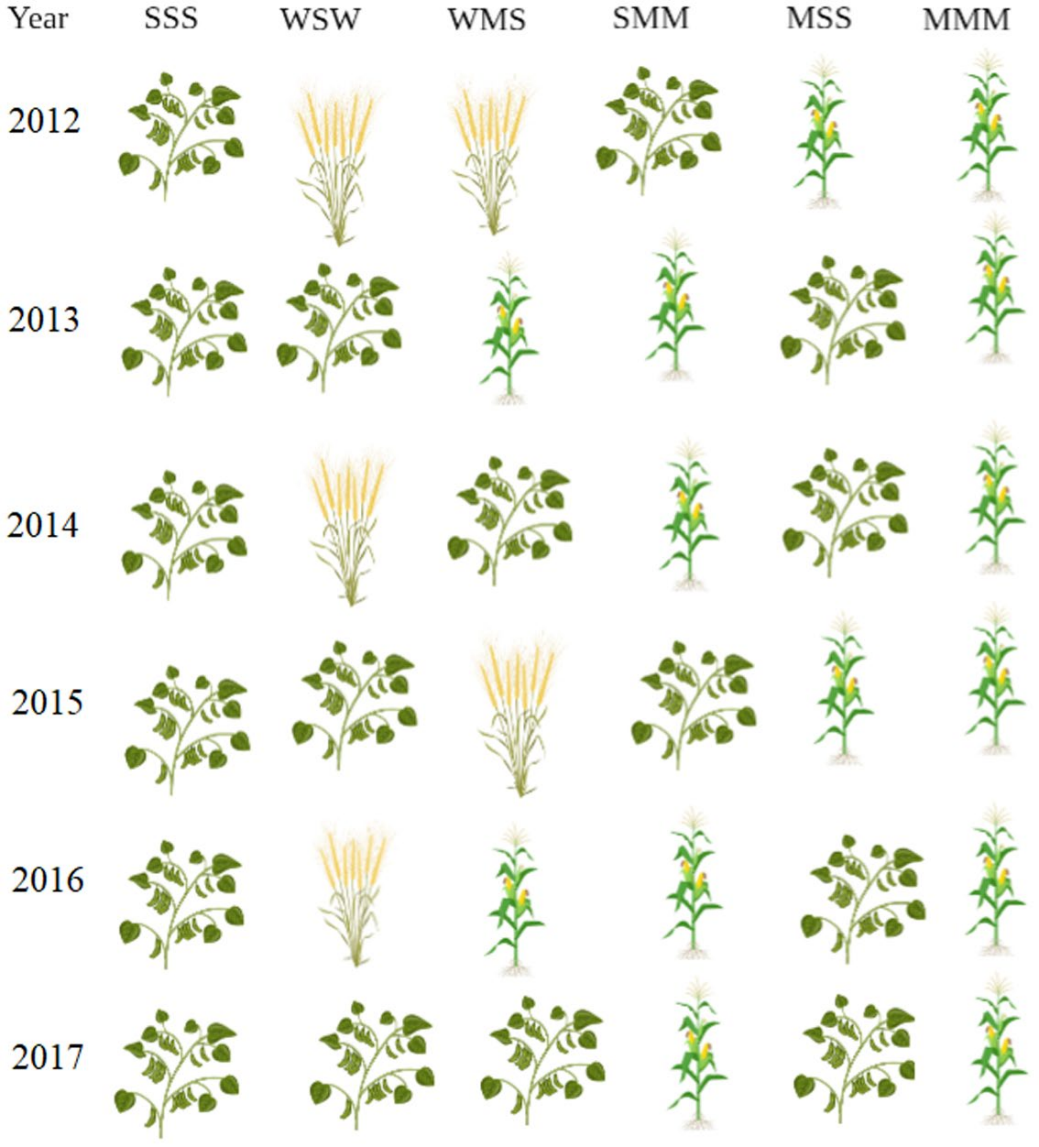
Crop rotation planting pattern once a year. Figure is created with BioRender.com.

The long-term field experiment was set up as a strip-plot design. The experiment includes six planting setups. Planting 1 setup includes only soybean (designated as sss), planting 2 setup includes wheat and soybean (designated as wsw), planting 3 setup includes wheat, maize and soybean (designated as wms), planting 4 setup includes soybean and maize (designated as smm), planting 5 setup includes maize and soybean (designated as mss) and planting 6 setup includes only maize (designated as mmm). Each type of cropping consists of three plots, there were 18 plots, each plot area of 87.75 m^2^. More details of the crop rotation system are mentioned in Figure 1. The application dosages of chemical fertilizers were set according to the soil testing and fertilizer recommendation and they were applied as base fertilizer when seeds were sown. For maize, soybean, and wheat, N-P_2_O_5_-K_2_O were 135-67.5-45, 50-60-45, and 75-80-45 kg/hm^2^, respectively.

### Crop harvest, sample collection, and soil properties analysis

After 5 years of crop cultivation, all crops in each plot were harvested and the yields were calculated with dry weight. Soil samples were taken from 0 to 15 cm soil depth in each plot after crop harvesting. Five subsamples were collected randomly from each plot and mixed evenly to form a bulked sample. A total of 18 samples (six treatments with three replicates) were collected and transported to the laboratory in a cooler with ice packs immediately. Each soil sample was passed through a 2-mm sieve, homogenized and plant roots and large rocks were removed. All the samples were divided into two parts: the first was air-dried, ground, and sieved (<0.25 mm) for soil physical and chemical properties analysis, and the second was stored at −70°C until DNA extraction.

Soil moisture (MO) was determined by gravimetrical methods. Soil pH was measured using a compound electrode at a 1:2.5 mass/volume soil-water suspension. Soil total organic carbon (TOC), total nitrogen (TN), total phosphorus (TP), total potassium (TK), available nitrogen (AN), available phosphorus (AP) and available potassium (AK) content were measured as described by Lu (2000).

### DNA extraction, PCR amplification, and sequencing

Total genomic DNA was extracted from the samples using the PowerSoil DNA isolation kit (MoBio) following the manufacturer’s instructions. The final DNA concentration and its purity was evaluated using NanoDrop 2000 UV VIS spectrophotometer (Thermo Scientific, Wilmington, United States). The DNA quality was checked using 1% agarose gel electrophoresis or by separating the DNA macromolecules in a matrix of agarose (Marcos et al., 2016).

The polymerase chain reaction (PCR) was carried out by the Phusion R High-Fidelity PCR Master Mix with GC Buffer (New England Biolabs) using specific primers with a barcode. The bacterial 16S rRNA genes with V3–V4 hypervariable regions were amplified with two primers 341F (5’-CCTAYGGGRBGCASCAG-3′) and 806R (5’-GGACTACNNGGGTATCTAAT-3′) (Yu et al., 2005). The fungal internal transcribed spacer (ITS) region was amplified using the primer pair ITS1F (5’-CTTGGTCATTTAGAGGAAGTAA-3′)/ITS2R (5’-GCTGCGTTCTTCATCGATGC-3′) (White et al., 1990). The PCR conditions used for the amplification of the16S rRNA genes and ITS region were as follows: 5 min of denaturation at 95°C, followed by 27 cycles of 30 s at 95°C, 30 s at 55°C for annealing, and 45 s at 72°C for elongation and ending with a final extension at 72°C for 10 min. The reaction mixture consists of 20 μl mixtures, each containing 4 μl of 5 × FastPfu Buffer, 2 μl of 2.5 mM dNTPs, 0.8 μl of each primer (5 μM), 0.4 μl of FastPfu polymerase, and 10 ng of template DNA (Tian et al., 2022). The obtained PCR products were checked on 2% agarose gel and further purified using an AxyPrep DNA Gel Extraction Kit (Axygen Biosciences, United States) and quantified using QuantiFluor™-ST (Promega, United States) according to the manufacturer’s instruction. The purified PCR products were pooled in equimolar ratios and paired-end sequenced using the HiSeq 2500 platform (Illumina, United States) at Biozeron Technology Co., Ltd. (Shanghai, China).

### Illumina sequence analysis

The raw FASTQ files were demultiplexed and quality-filtered using Trimmomatic (a flexible read trimming tool) (Bolger et al., 2014) and then the sequences data were merged using the FLASH (Fast Length Adjustment of Short reads) tool (Magoč and Salzberg, 2011). Chimeric sequences were identified and removed by applying UCHIME (Edgar et al., 2011). Operational taxonomic units (OTUs) were clustered at a 97% sequence identity cut-off *via* UPARSE (Edgar, 2013). The OTU representative sequences of bacteria and fungi were identified taxonomically using the Ribosomal Database Project (RDP) Classifier (Wang et al., 2007) and UNITE databases (Nilsson et al., 2018). The detected taxa were averaged across three biological replicates.

The QIIME 2 software was used to calculate the observed species (Bolyen et al., 2019), and the R software (version 2.15.3)^2^ was used to draw the species accumulation curve. Bacterial and fungal alpha diversity including richness estimators (Chao and ACE) and diversity indices (Shannon and Simpson) were calculated using Mothur v.1.41.1^3^. The principal coordinate analysis (PCoA) map was drawn by the R software (see footnote 2) using the WGCNA, stats, and ggplot2 software packages (Wickham, 2009). Molecular ecological network analyses were conducted to reveal the variations in the interactions between phylotypes responding to different cropping sequences, according to an online pipeline (Zhou et al., 2010; Xiao et al., 2022). The correlation network was visualized using the R package igraph. FUNGuild was used to predict fungal function based on ITS sequence data (Nguyen et al., 2016). The functional composition prediction was performed using PICRUSt (phylogenetic investigation of communities by reconstruction of unobserved states) (Langille et al., 2013).

The raw sequences for all samples have been deposited in the NCBI Sequence Read Archive (SRA) database with accession numbers PRJNA883616 and PRJNA884493 for bacteria and fungi, respectively.

### Statistical analysis

The soil properties (including pH value, moisture, total carbon, total nitrogen, and total phosphorus, total potassium, available nitrogen, available phosphorus and available potassium in soil) were analysed by one-way analysis of variance (ANOVA) in SPSS v22.0. The relationship between the alpha diversity of soil bacteria and fungi and soil properties was examined using corrplot in R software (version 4.1.0). Community composition was visualised using Redundancy analysis (RDA) with soil properties fitted onto the RDA ordination as vectors.

## Results and discussion

### Crop sequence affected soil physicochemical properties and crop yields

Experiencing the 5 years monoculture and rotation experiment, the soil properties such as pH, water content and total organic carbon, potassium, phosphorus, and nitrogen were significantly changed (Supplementary Table S1). All crop rotations increased soil TOC, TN and AN content. Soil TOC and TN were similar between smm and mss, but AN was higher in mss, AP and AK was higher in smm. In our design of planting patterns, compared with monocultures, soybean and maize increased the yield regardless of crop combinations and rotation sequence. Soybean-maize rotation (mss) increased soybean yield by 11.27% compared with continuous soybean cropping (sss). Compared with continuous cropping (mmm), wms and smm increased maize yield by 1.64 and 23.06%, respectively (Supplementary Table S2), and smm was the most significant. Obvious changes related to soil pH was noticed in wms and wsw planting systems, wms can maintain an almost neutral soil pH, however, wsw made soil pH similar to soybean monoculture (sss).

Earlier studies suggest that continuous single cropping has resulted in the deterioration of soil chemical properties and nutrient imbalance (Liu et al., 2012; Liu Z. et al., 2020) and crop rotation has a significant impact on soil pH (Karlen et al., 2006; Liu Z. et al., 2020). In the present study, soybean and maize rotation in different ways can significantly increase soil pH compared to soybean and wheat rotation. Although we were unable to compare soybean yields under the wheat-soybean rotation (wsw) pattern, however, based on the data from soil nutrients, we predicted that soybean yield may not exceed smm and smm in the next year, moreover wsw rotation made soil pH similar to soybean monoculture (sss), and this type of rotation does not play any role in preventing soil acidification. In a study, Martens (2000) reported that crop rotation significantly increased soil organic carbon content, while it did not happen in monoculture soybeans. Similarly, our findings showed that different crop rotations increased total organic carbon content compared to monocultures (sss and mmm), which decreased total potassium, phosphorus, and nitrogen content (Supplementary Table S1).

### Cropping sequences changed the diversity and composition of the soil microbial community

Using the Illumina HiSeq 2500 platform, a total of 799,070 bacterial and 790,459 fungal high-quality clean reads were obtained from the six cropping patterns and grouped into 7,564 OTUs for the bacteria, and 2,895 OTUs for the fungi. The alpha diversity (Supplementary Figures S1A,B) result showed that the rarefaction curves approached a plateau, indicating that the number of OTUs was sufficient to reveal the authentic bacterial and fungal communities within each sample.

Alpha diversity of bacterial and fungal community among all cropping sequences are observed in Supplementary Table S3. The number of observed bacterial species was greater in smm planting setup when compared with the other planting setup, mss planting showed the least number of observed bacterial species (Supplementary Figure S1C). In contrast to the number of observed bacterial species, the number of observed fungal species was greater in mss planting setup (Supplementary Figure S1D). Compared with continuous planting of maize (mmm), planting soybean could significantly increase the soil bacterial richness, which was the highest in smm, but there was no significant difference compared to other soybean cropping sequences. There was a significant difference in Simpson index and Shannon index between mss and wsw, wms, and mmm in bacteria, but no significant difference in Simpson index in fungi, and significant difference in Shannon index between smm and wsw and mmm. According to the findings, different cropping systems had different effects on bacterial and fungal communities, especially between rotation and monoculture.

Some studies have shown that intensive land use can lead to homogenization of soil microbial communities, reduction of taxa and/or dominance of nutrient groups, and decline of overall diversity (Banerjee and van der Heijden, 2023). In this study, there was no significant difference in Chao and ACE among soybean varieties planted in different sequences, including continuous cropping and rotation, which was consistent with findings in previous studies that legume crop rotation had little effect on soil bacterial richness perhaps due to low diversity of the host-specific microbes associated with legumes relative to free-living microorganisms (Venter et al., 2016; Paungfoo-Lonhienne et al., 2017). In two different rotation patterns (smm and mss; designed for soybean and maize) mss significantly increased bacterial diversity while decreased fungal diversity. Similarly, Neupane et al. (2021) evaluated long-term crop rotation effect on subsequent soybean yield, they found significant differences in the alpha diversity for root-associated bacterial and fungal communities among the four treatments (continuous corn, corn/corn/soybean, corn/soybean, and soybean/corn). In addition, some report suggests that alpha diversity of both bacterial and fungal communities was lower in continuous single cropping when compared to different plant crop rotation (Liu Z. et al., 2020). Similar results were obtained in this study, especially the α-diversity of fungi, which was lowest in sss.

**Figure 2:**
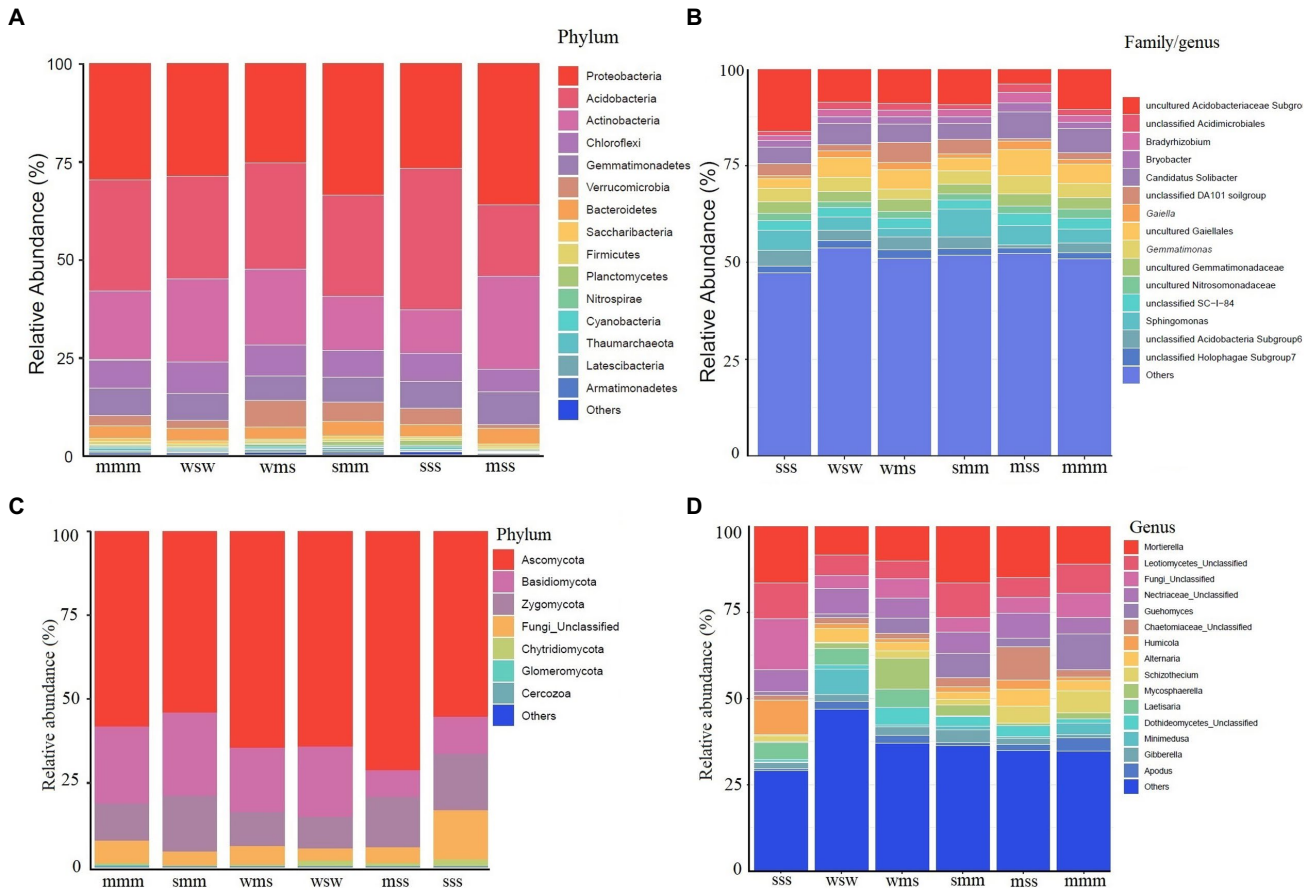
Relative abundance of microbial communities in different planting systems: **(A, C)** phylum level, **(B, D)** family/genus level. The x-axis represents samples and the y-axis represents relative abundance presented as a percentage.

The composition of the soil microbial community showed that the top fifteen phyla in all samples were *Proteobacteria*, *Acidobacteria*, *Actinobacteria*, *Chloroflexi*, *Gemmatimonadetes*, *Verrucomicrobia*, *Bacteroidetes*, *Saccharibacteria*, *Firmicutes*, *Planctomycetes*, *Nitrospirae*, *Cyanobacteria*, *Thaumarchaeota*, *Latescibacteria*, and *Armatimonadetes* but their proportion varied (Figure 2A). Researchers found that the phyla *Acidobacteria* and *Proteobacteria* make up the majority of the soil bacterial community in crop rotation systems (Chamberlain et al., 2020). In the present study also a similar microbial abundance was found. Analogously, Bolaji et al. (2021) reported *Actinobacteria*, *Proteobacteria*, and *Acidobacteria* as the dominant taxa in canola, corn, and soybean crop rotation planting systems.

When compared to the other crop rotation systems, the relative abundance of *Proteobacteria* was higher in the maize and soybean (mss and smm) crop rotation systems, *Thaumarchaeota* was noticed in some planting systems (Figure 2A). Recently, the abundance of *Thaumarchaeota* in the plant rhizosphere was detected and suggested their role in nitrogen cycling (Zhang et al., 2020). The sss planting system had a high abundance of *Acidobacteria* while mss planting system had a lower abundance when compared to other planting systems. *Verrucomicrobia* abundance was noticed in wms planting system. A similar study was conducted to evaluate the impact of crop rotation management of soybean-wheat and maize-wheat on microbial diversity. It was noticed that soybean-wheat rotation increased the relative abundances of *Firmicutes* and *Bacteroidetes* and reduced *Actinobacteria*, *Verrucomicrobia*, and *Chloroflexi* compared to maize-wheat rotation (Yu et al., 2021). Strong positive correlations were observed between Proteobacteria and TN, AK; *Actinobacteria Verrucomicrobia* and *Chloroflexi* and AP. These taxa play a major role in soil nutrient cycling, to improve nutrient uptake and productivity in crop rotation.

At the genus level, the majority of the OTUs were unclassified, suggesting they might be novel candidates (Figure 2B). The relative abundance of *Acidobacteriaceae* was high in sss planting system, which could due to low soil pH (Supplementary Table S1). The abundance of “*Candidatus* Solibacter” was noticed in all planting systems. A little information was available regarding “*Candidatus* Solibacter” and our future research will focus on its role in soybean crop rotation. Taxa like *Gemmatimonas* and *Gaiella* were reported to be abundant under standard nitrogen fertilization (Meier et al., 2021). *Gemmatimonas* and *Gaiella* were also noticed in the present study planting systems (Figure 2B). Such taxa were reported to be key players in nitrification and nitrogen assimilation (Dong et al., 2016; Morrissey et al., 2018).

The abundance of fungi was less when compared with bacteria in all planting systems. The top phyla were *Ascomycota*, *Basidiomycota*, *Zygomycota*, *Chytridiomycota*, *Glomeromycota*, and *Cercozoa* (Figure 2C). Similarly, Narayana et al. (2022) noticed *Ascomycota* and *Basidiomycota* abundance in maize and soybean rotation. *Mortierella* abundance was noticed in almost all planting systems (Figure 2D). Members of the genus *Mortierella* have an impact on the control of plant diseases. It has been demonstrated that some *Mortierella* species were effective at preventing clubroot disease (Narisawa et al., 1998). *Guehomyces* abundance was noticed in maize planting system when compared to other plant crop rotations (Figure 2D) indicating plant-specific abundance. Detailed fungal abundance in different planting systems is depicted in Figure 2.

### Soil microbial communities are closely related to soil properties and crop yields

**Figure 3:**
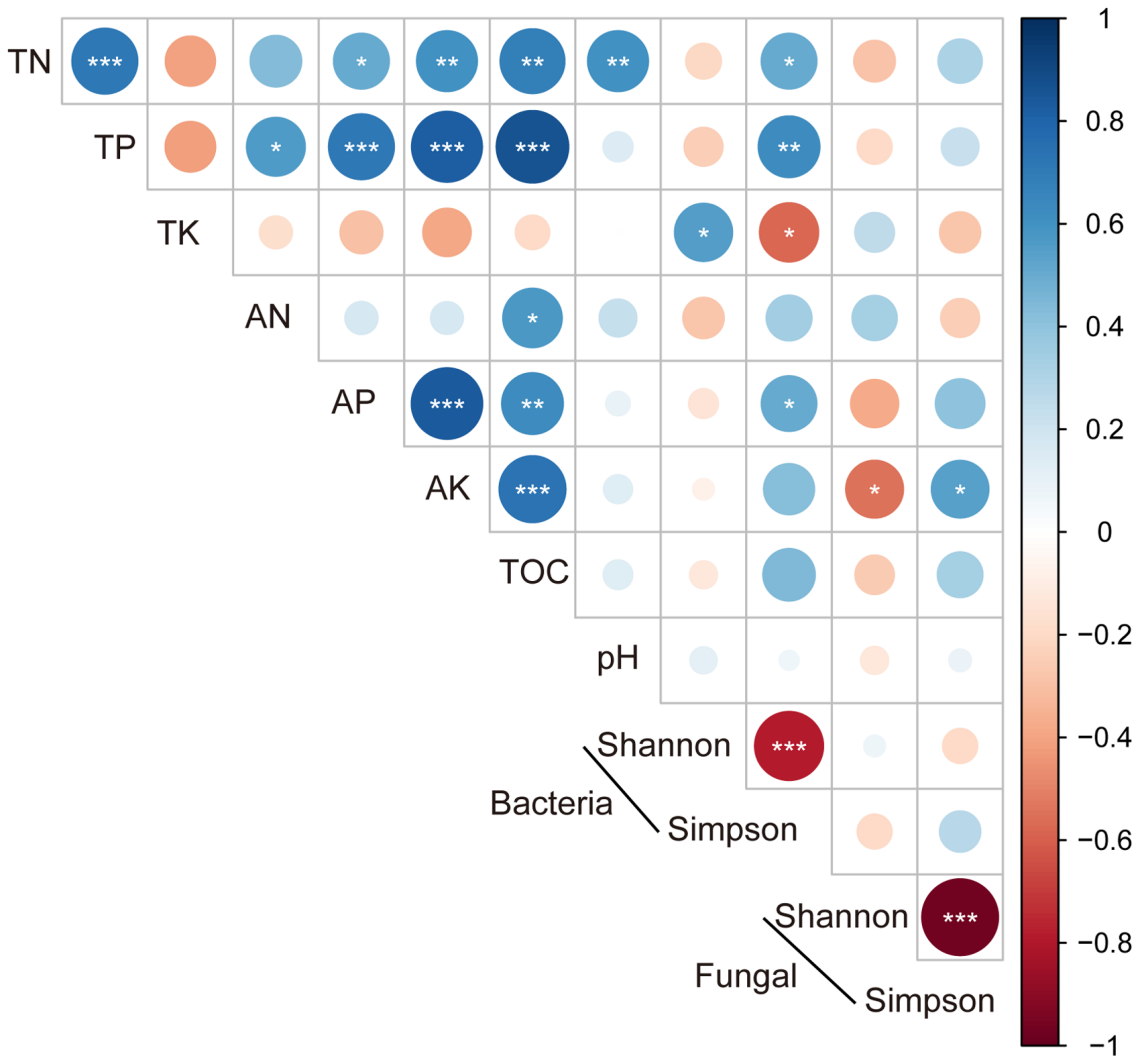
The relationship between the alpha diversity and soil properties (*, ≤0.05; **, ≤0.01; ***, ≤0.001).

The linear relationships between microbial alpha diversity and soil properties revealed that the bacterial Shannon index was significantly and positively correlated with soil TK content (*p* < 0.05), and Simpson index was significantly and positively correlated with TN (*p* < 0.05) and AP (*p* < 0.01). The Shannon index and Simpson index of fungi were significantly and positively and negatively correlated with AK content (0.05 level), respectively, but had no correlation with other soil properties (Figure 3).

**Figure 4:**
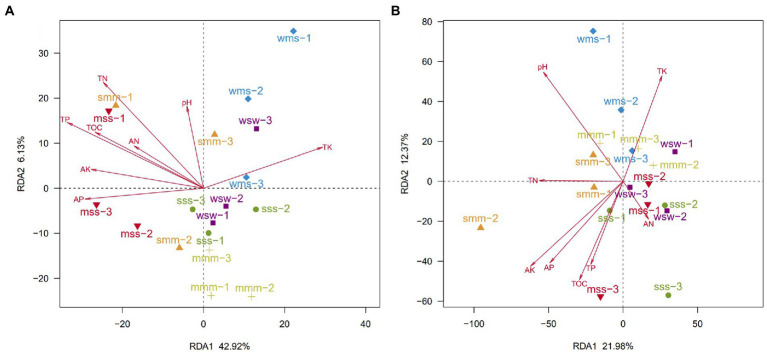
Redundancy analysis (RDA) of bacterial **(A)** and fungal **(B)** community compositions and soil chemical properties in all cropping sequences. (OTUs with a relative abundance of more than 0.01%).

The RDA axis separated the soil bacterial communities of continuous and rotation cropping systems, the results also showed that soil total organic carbon (TOC), total nitrogen (TN) and total phosphorous (TP) had significant effects on soil bacterial community structure of soybean and maize in different cropping sequence (Figure 4A). However, the change of soil pH caused by different types of crops and their different cropping sequences are the main factor affecting the change in fungal community (Figure 4B). This was in agreement with findings by Ai et al. (2018) that bacterial community structure was more sensitive to changes in soil nutrient levels induced by fertilization than fungal community structure.

**Figure 5:**
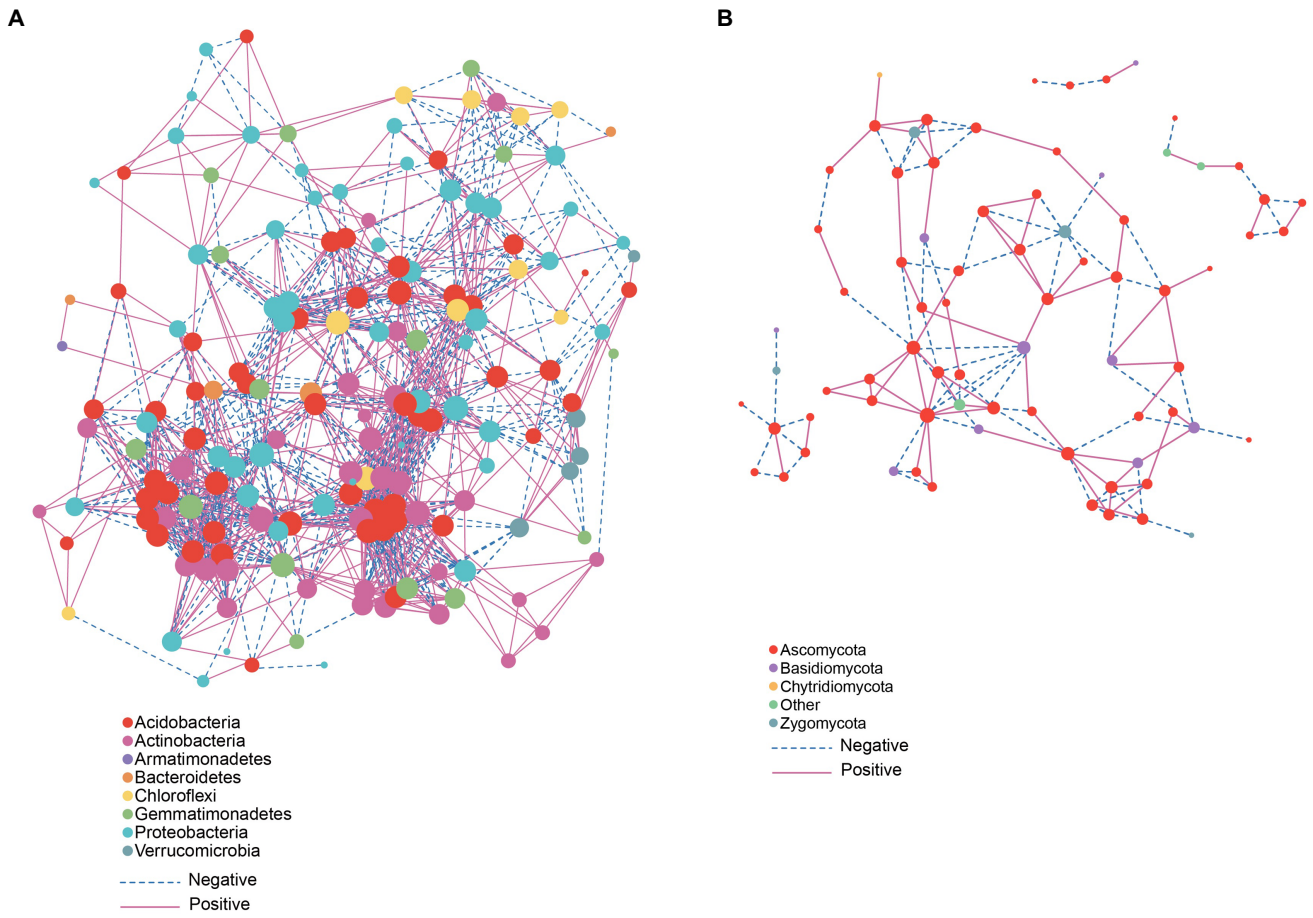
Network-based analysis: **(A)** Bacterial communities and **(B)** fungal communities.

**Figure 6:**
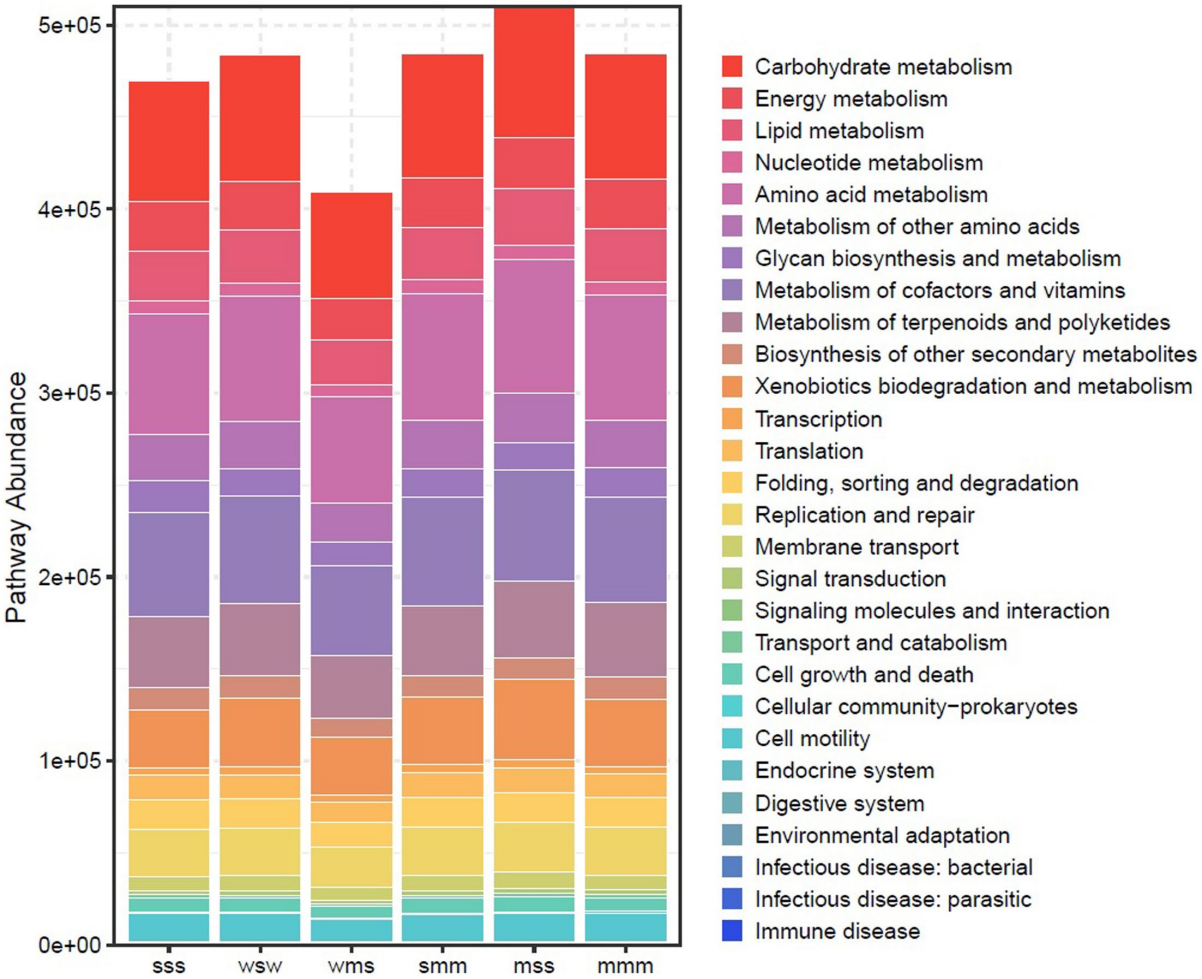
PICRUSt based predicted functional composition.

Microorganisms participate in multiple inter- and intra- kingdom interactions. In particular, network-based techniques have been proven useful in interpreting complex microbial interaction patterns (Matchado et al., 2021). Network-based analysis suggests that phyla *Acidobacteria* and *Actinobacteria* showed a positive correlation with other members (Figure 5A) while *Proteobacteria* in most cases showed a negative correlation with other members. In fungal network analysis, *Ascomycota* showed a positive correlation with other members (Figure 5B). We used PICRUSt to predict the functional composition. The PICRUSt result (Figure 6) showed the presence of various metabolic pathways. The planting setup mss showed the highest pathway abundance while wsw, smm and mmm showed similar pathway abundance (Figure 6).

**Figure 7:**
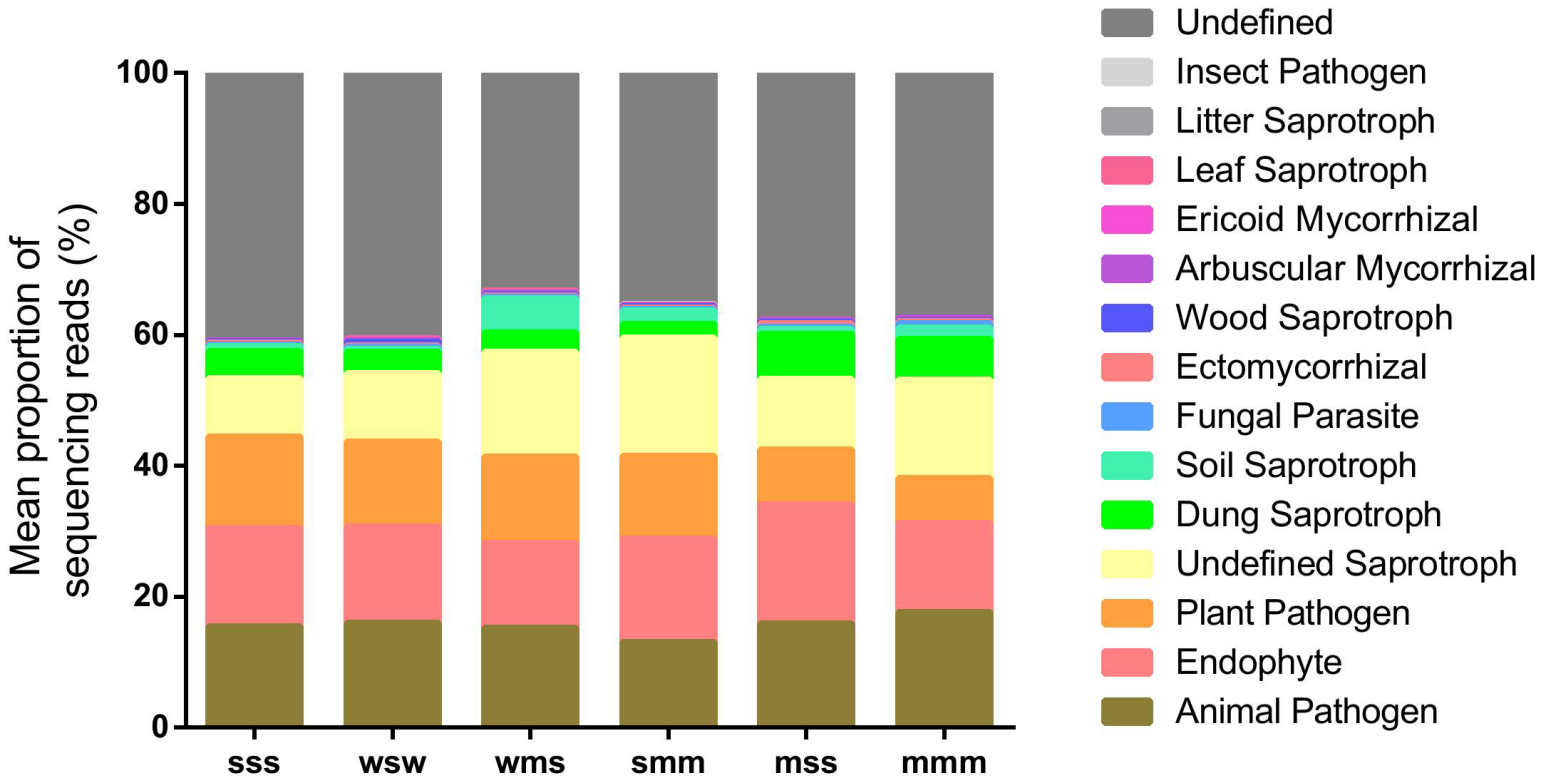
Proportion of fungal taxa based on functional annotation by FUNGuild (spearman correlation coefficient).

Moreover, we used FunGuild (spearman correlation coefficient) to perform a functional classification of fungal ITS data, the results of which are shown in Figure 7. Different cropping sequences affected the mean proportion of animal and plant pathogens in soil, it was mss < mmm < smm < wsm < wsw < sss. Some beneficial microorganisms, such as like endophyte, ectomycorrhizal and arbuscular mycorrhizal, were found to have the highest proportion in mss, followed by smm. The wms had the lowest proportion of beneficial microorganisms in all cropping patterns, probably due to the low soil pH in it.

One of the most well-known examples of plant beneficial microorganisms were the mycorrhizal fungi, which form symbiotic associations with nearly 90% of land plants, including many crops (van der Heijden et al., 2015). These fungi help plants obtain water and essential micronutrients and macronutrients from the soil to promote plant growth (Hoeksema et al., 2010). These findings in our study suggest that soybean-maize rotation (mss and smm) can reduce the potential risk of soil-borne animal and plant diseases, increase beneficial functional groups, and maintain a healthy soil ecosystem.

## Conclusion

Crop rotation is one of the effective agricultural management approaches used by farmers to avoid soil degradation and yield decline. The present study evaluated six crop sequences (sss, wsw, wms, smm, mss, and mmm) performed for 5 years. The crop rotation affected the soil physicochemical parameters, which showed soybean and maize rotation (smm and mss) can increase soil organic matter, nitrogen, phosphorus and potassium, which is helpful to increase the yield of soybean and maize. The cropping sequence also changed the diversity and composition of soil microbial community. However, the effects of different cropping sequences on soil bacterial and fungal diversity were not significant. Functional and microbial diversity analysis showed that the present crop rotation planting system showed the presence of nitrogen-fixing bacteria and taxa that inhibit plant pathogens. Interestingly, the highest maize production was noticed in smm planting system, smm can help soil to obtain a lower proportion of pathogens and a higher proportion of beneficial microorganisms. Finally, our findings might be used to guide decision-making for yearly crop and soil management strategies in major grain-producing regions of northeast China.

## Data availability statement

The datasets presented in this study can be found in online repositories. The names of the repository/repositories and accession number(s) can be found at: https://www.ncbi.nlm.nih.gov/, PRJNA883616 and https://www.ncbi.nlm.nih.gov/, PRJNA884493.

## Author contributions

LS and SW designed the research and project outline. YS, Z-HL, P-JJ, WW, Y-ML, and K-KW performed the DNA extraction and raw data analysis. MN and AB drafted the manuscript. X-YC and DW supervised the study. All authors contributed to the article and approved the submitted version.

## Funding

This work was supported by National Key R&D Program of China MOST (no. 2021YFD1500300), China Agriculture Research System of MOF and MARA (CARS-04-PS17), Heilongjiang Science and Technology Project (2021ZXJ05B011), Heilongjiang Academy of Agricultural Sciences Research Project (2020FJZX001 and 2021QKPY008), and UNDP Project (cpr/21/401).

## Conflict of interest

The authors declare that the research was conducted in the absence of any commercial or financial relationships that could be construed as a potential conflict of interest.

## Publisher’s note

All claims expressed in this article are solely those of the authors and do not necessarily represent those of their affiliated organizations, or those of the publisher, the editors and the reviewers. Any product that may be evaluated in this article, or claim that may be made by its manufacturer, is not guaranteed or endorsed by the publisher.

## Footnotes

^1^ https://heilongjiang.dbw.cn/system/2018/03/01/057939012.shtml

^2^ http://www.r-project.org/

^3^ http://www.mothur.org/wiki
