# Supplementary material for: The shift of soil microbial community induced by cropping sequence affect soil properties and crop yield
Source: Front Microbiol. 2023 Feb 16;14:1095688. doi: 10.3389/fmicb.2023.1095688 (PMC10004276; doi:10.3389/fmicb.2023.1095688)
Supplement: Supplementary file 1 [file Data_Sheet_1.docx]

# Supplementary materials

# The shift of soil microbial community induced by cropping sequence affect soil properties and crop yield

# Lei Sun^1,2^† Shuang Wang^2^†, Manik Prabhu Narsing Rao^3^, Yu Shi^4^, Zhen-Han Lian^3^, Pin-Jiao Jin^2^, Wei Wang^2^, Yu-Mei Li^2^, Kang-Kang Wang^2^, Aparna Banerjee^6^, Xiao-Yang Cui^1^* & Dan Wei^5*^

^1^Key Laboratory of Sustainable Forest Ecosystem Management-Ministry of Education, School of Forestry, Northeast Forestry University, Harbin 150040, PR China

^2^Heilongjiang Academy of Black Soil Conservation & Utilization, Harbin 150086, PR China

^3^Programa de Doctorado en Ciencias Aplicadas, Universidad Autónoma de Chile, Talca, 3460000, Chile

^4^State Key Laboratory of Crop Stress Adaptation and Improvement, School of Life Sciences, Henan University, Kaifeng 475000, China

^5^Institute of Plant Nutrition and Resources, Beijing Academy of Agriculture and Forestry Sciences, Beijing 100097, PR China

^6^Centro de Investigación de Estudios Avanzados del Maule (CIEAM), Vicerrectoría de Investigación y Postgrado, Universidad Católica del Maule, Talca, 3466706, Chile

***Correspondence:**

Xiaoyang Cui*: [c_xiaoyang@126.com](mailto:c_xiaoyang@126.com)

Dan Wei^*^: [wd2087@163.com](mailto:wd2087@163.com)

†These authors contributed equally to this work

**
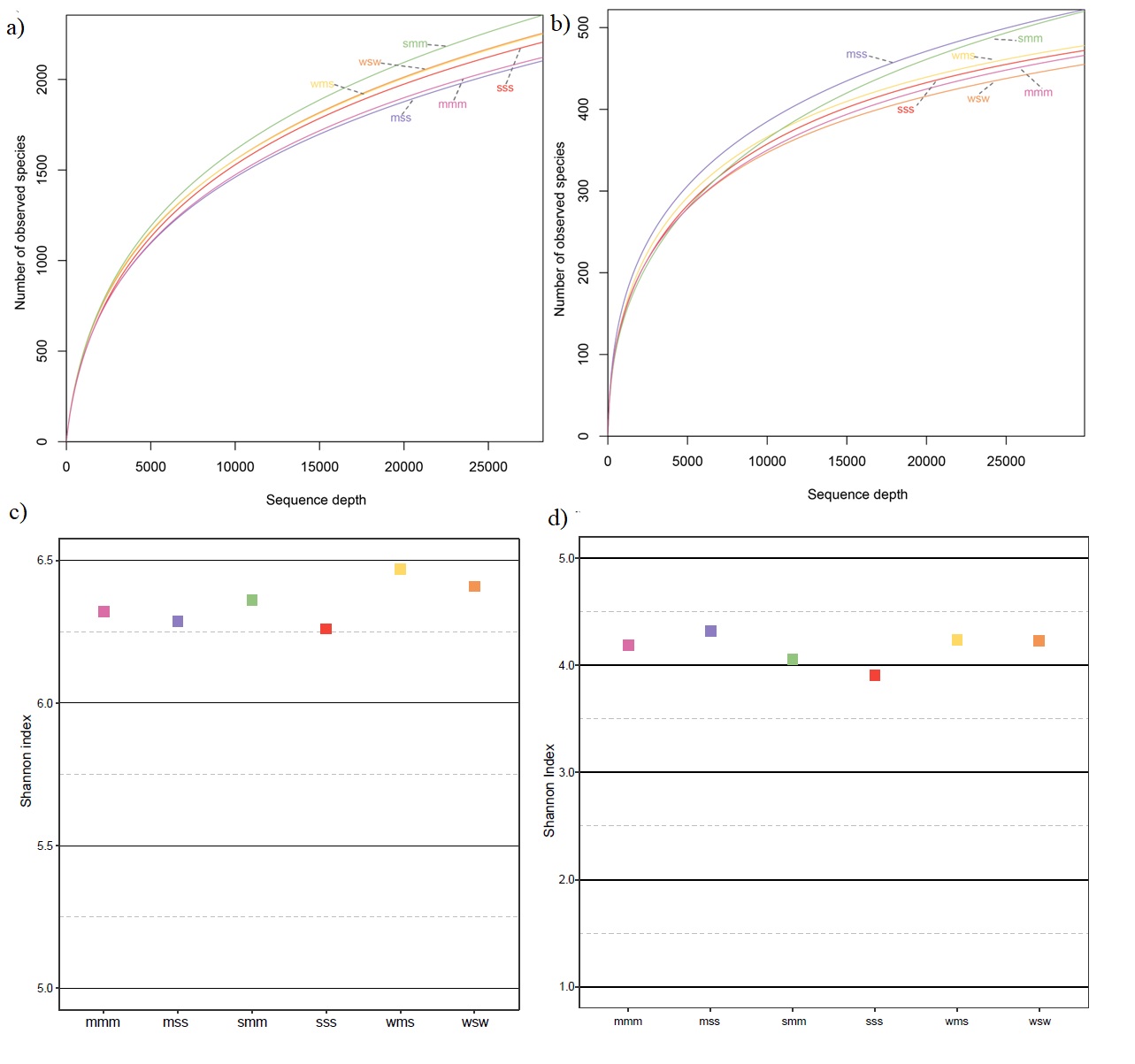
**

**Supplementary Figure S1.** a) alpha diversity showing bacterial OTUs per sequence number; b) alpha diversity showing fungal OTUs per sequence number; c) bacterial Shannon's diversity index; d) fungal Shannon's diversity index.

Table S1 Soil properties across different cropping sequences.

| Crop sequence | pH | MO (%) | TOC (g🞄kg^-1^) | TN (%) | AN (mg🞄kg^-1^) | TP (%) | AP (mg🞄kg^-1^) | TK (%) | AK (mg🞄kg^-1^) |
| --- | --- | --- | --- | --- | --- | --- | --- | --- | --- |
| sss | 6.03±0.11b | 18.76±3.56bc | 33.90±0.26ac | 0.20±0.01a | 164.17±11.35a | 0.08±0.00d | 25.67±0.23ab | 2.52±0.09ab | 183.67±8.08c |
| wsw | 6.04±0.05b | 15.17±2.20ac | 34.67±1.77c | 0.20±0.01a | 173.43±19.15a | 0.08±0.00d | 24.53±0.47a | 2.55±0.01ab | 163.00±4.58a |
| wms | 6.91±0.23c | 16.14±2.16ac | 34.73±2.20c | 0.22±0.01b | 177.13±9.83ab | 0.08±0.00d | 24.30±2.26a | 2.58±0.02b | 170.33±6.81a |
| smm | 6.64±0.09ac | 20.41±1.78b | 37.60±0.89b | 0.22±0.01b | 175.47±15.04a | 0.08±0.00c | 30.00±2.60b | 2.52±0.06ab | 213.00±13.23b |
| mss | 6.61±0.23ac | 21.82±4.14b | 38.87±1.21b | 0.23±0.01b | 197.57±8.12b | 0.08±0.00b | 27.93±2.02b | 2.47±0.07a | 201.67±4.16b |
| mmm | 6.60±0.17a | 12.68±1.31a | 31.53±0.91a | 0.20±0.01a | 173.00±0.00a | 0.07±0.00a | 24.93±0.57a | 2.53±0.05ab | 162.33±12.10a |

Note: Values represent the means and standard deviations and followed by different lowercase letters are significantly different (LSD, *P* < 0.05).

Table S2 Crop yield from different cropping sequences.

| Crop sequence | Harvested crops | Yield per plot (kg🞄ha^-1^) | Variables (x') after Z-score | Significance of difference | Crop yield (kg🞄ha^-1^) |
| --- | --- | --- | --- | --- | --- |
| sss | Soybean | 1743.95 | -1.0803 | a | 1709.68±34.27a |
|  |  | 1675.4 | -1.10075 |  |  |
|  |  | 1709.68 | -1.09052 |  |  |
| wsw | Wheat | 2988.56 | -0.70909 | b | 2948.18±50.05b |
|  |  | 2963.8 | -0.71647 |  |  |
|  |  | 2892.18 | -0.73783 |  |  |
| wms | Maize | 7910.34 | 0.75888 | c | 8024.52±119.96c |
|  |  | 8013.7 | 0.7897 |  |  |
|  |  | 8149.52 | 0.83021 |  |  |
| smm | Maize | 9726.78 | 1.30064 | d | 9715.81±161.46d |
|  |  | 9871.5 | 1.34381 |  |  |
|  |  | 9549.14 | 1.24766 |  |  |
| mss | Soybean | 1901.72 | -1.03324 | a | 1902.41±39.66a |
|  |  | 1863.1 | -1.04476 |  |  |
|  |  | 1942.41 | -1.02111 |  |  |
| mmm | Maize | 7890.97 | 0.7531 | c | 7895.30±331.19c |
|  |  | 7566.3 | 0.65626 |  |  |
|  |  | 8228.64 | 0.85381 |  |  |

Note: The data of crop yield represent the mean ± S.D (n = 3). Different lowercase letters within a column indicate significant differences among treatments (LSD, *P* < 0.05). Z-score: Zero-mean normalization. The processed data were normal distribution, meaning that about half of the variables were less than 0, the other half were more than 0, the variables had a mean of 0 and a standard deviation of 1, the range of variable is -1≤ x' ≤1.

Table S3 Alpha diversity of soil bacteria and fungi in different cropping sequences.

| Crop sequence | Bacteria | | | | |  | Fungi | | | | |
| --- | --- | --- | --- | --- | --- | --- | --- | --- | --- | --- | --- |
|  | Chao | ACE | Shannon. | 1/Simpson. | OTUs |  | Chao | ACE | Shannon. | 1/Simpson. | OTUs |
| sss | 2938.33±112.63a | 2938.67±126.34a | 6.35±0.08ab | 195.98±44.07ac | 2219.00±52.72a |  | 537.33±2.08ab | 536.33±1.53ab | 4.09±0.16ab | 28.03±7.59ab | 468.00±5.29a |
| wsw | 2991.00±133.13a | 2994.33±96.26a | 6.38±0.03b | 219.07±5.46ab | 2225.33±54.86a |  | 517.67±23.86b | 508.33±17.21b | 4.26±0.05a | 31.21±1.05ab | 454.33±3.06a |
| wms | 3039.67±24.01a | 3018.00±44.64a | 6.43±0.05b | 249.63±24.73b | 2238.67±25.01a |  | 548.33±46.01ab | 548.33±46.01abc | 4.23±0.23a | 34.09±9.46ab | 490.67±41.48a |
| smm | 3111.33±116.45a | 3062.67±113.46a | 6.40±0.04b | 162.20±40.37ac | 2287.00±113.46a |  | 595.67±72.14ac | 590.67±66.89ac | 3.88±0.32b | 22.56±7.49a | 465.67±60.17a |
| mss | 2933±116.45a | 2938.00±122.48a | 6.28±0.03a | 144.97±24.50c | 2185.67±73.36a |  | 612.33±34.08c | 613.00±29.14c | 4.20±0.14a | 28.59±6.32ab | 507.00±14.53a |
| mmm | 2264±410.67b | 2348.33±391.13b | 6.34±0.05ab | 207.41±12.83ab | 2020.33±93.85b |  | 520.67±20.11ab | 516.67±27.10b | 4.29±0.09a | 35.16±6.27b | 456.00±17.32a |

Note: OTUs, operational taxonomic units (97% similarity); Values represent the means and standard deviations and followed by different lowercase letters are significantly different (LSD, *P* < 0.05)
